# Radixin Relocalization and Nonmuscle *α*-Actinin Expression Are Features of Remodeling Cardiomyocytes in Adult Patients with Dilated Cardiomyopathy

**DOI:** 10.1155/2020/9356738

**Published:** 2020-07-22

**Authors:** Ayse Cetinkaya, Benedikt Berge, Bedriye Sen-Hild, Kerstin Troidl, Praveen Gajawada, Natalia Kubin, Klaus Valeske, Dietmar Schranz, Hakan Akintürk, Markus Schönburg, Thomas Kubin, Yeong-Hoon Choi, Manfred Richter

**Affiliations:** ^1^Department of Cardiac Surgery, Kerckhoff Heart Center, Benekestrasse 2-8, Bad Nauheim 61231, Germany; ^2^Justus-Liebig-University Gießen, Campus Kerckhoff, Bad Nauheim, Germany; ^3^Pediatric Heart Center, Justus Liebig University, Feulgenstrasse 10-12, Giessen 35392, Germany; ^4^Max Planck Institute for Heart and Lung Research, Benekestrasse 2-8, Bad Nauheim 61231, Germany; ^5^German Center for Cardiovascular Research (DZHK), Partner Site RhineMain, Frankfurt/Main, Germany

## Abstract

**Background:**

Pediatric patients show an impressive capacity of cardiac regeneration. In contrast, severely deteriorated adult hearts do usually not recover. Since cardiac remodeling—involving the expression of fetal genes—is regarded as an adaptation to stress, we compared hearts of adult patients suffering from dilated cardiomyopathy (DCM) with remodeling of cultured neonatal (NRC) as well as adult (ARC) rat cardiomyocytes and the developing postnatal myocardium.

**Methods:**

NRC and ARC were stimulated with serum and cardiac morphogens derived from DCM hearts. Protein synthesis (PS) as well as protein accumulation (PA) was measured, and cell survival was determined under ischemic conditions. Fetal markers were investigated by Western blot. Biomarkers of remodeling were analyzed in controls, DCM, and 2- to 6-month-old children with tetralogy of Fallot as well as in neonatal and adult rats by immunofluorescence.

**Results:**

In NRC, serum and morphogens strongly stimulated PS and PA and the reestablishment of cell-cell contacts (CCC). In ARC, both stimulants increased PS and CCC, but PA was only elevated after serum stimulation. In contrast to serum, morphogen treatment resulted in the expression of fetal genes in ARC as determined by nonmuscle *α*-actinin-1 and *α*-actinin-4 expression (NM-actinins) and was associated with increased survival under ischemia. NM-actinins were present in cardiomyocytes of DCM in a cross-striated pattern reminiscent of sarcomeres as well as in extensions of the area of the intercalated disc (ID). NM-actinins are expressed in NRC and in the developing heart. Radixin staining revealed remodeling of the area of the ID in DCM almost identical to stimulated cultured ARC.

**Conclusions:**

Remodeling was similar in ARC and in cardiomyocytes of DCM suggesting evolutionary conserved mechanisms of regeneration. Despite activation of fetal genes, the atrophy of ARC indicates differences in their regenerative capacity from NRC. Cardiac-derived factors induced NM-actinin expression and increased survival of ischemic ARC while circulating molecules were less effective. Identification of these cardiac-derived factors and determination of their individual capacity to heal or damage are of particular importance for a biomarker-guided therapy in adult patients.

## 1. Introduction

The function of the heart is not only the continuous supply of oxygen and nutrients but also the removal of degradation products such as CO_2_ by pumping blood through the circulatory system. When the healthy heart is challenged by increased physiological workload, for example, during pregnancy or in high-performance athletes, the myocardium reacts with hypertrophy but sustains cardiac architecture as well as the differentiation status of the ventricle. However, under chronic conditions such as dilated cardiomyopathy, the “status quo” is disturbed and ventricular remodeling occurs which affects the amount and the composition of proteins in cardiac cells and the extracellular matrix. Often, inflammatory processes even at a low grade might continuously contribute to cardiac remodeling by changing the level of cytokines and leukocyte subsets in the tissue as well as in the circulation [1–4].

Cardiac remodeling has been defined in a consensus paper from an international forum in 2000 as “genome expression, molecular, cellular and interstitial changes that are manifested clinically as changes in size, shape and function of the heart after cardiac injury” [5]. Importantly, the etiology of adult cardiac remodeling might significantly differ between various heart diseases, but the transition from compensatory processes to heart failure shares many common pathways [5–10]. By keeping this in mind, it becomes clear that strategies to determine the mechanism behind the transition to heart failure, its therapy, and risk stratification are still poorly established and require more basic knowledge [11].

One common pathway in the development of heart failure is the reactivation of fetal genes. Activation of fetal genes involves a switch in the protein expression from adult to fetal/neonatal isoforms such as smooth muscle *α*-actin and myosin heavy chains, but also fetal nonisoforms like the ERM protein moesin are reexpressed in cardiomyocytes [8, 12, 13]. However, despite the activation of several evolutionary conserved survival pathways under pathological conditions, the regenerative capacity of the adult myocardium still remains poor. When the heart fails to meet metabolic demands, patients develop chronic heart failure from which adult people usually do not recover. In contrast, there are a number of clinical examples showing that young children have an astonishing regenerative capacity. Children with anomalous origin of the left coronary artery from the pulmonary artery with severely dilated hearts regenerate after surgical correction [14]. Pediatric patients with univentricular hearts recover after volume unloading [15], and children with dilated cardiomyopathy are able to regenerate spontaneously from severely impaired heart function [16]. Thus, activation of fetal genes might be an incomplete attempt of adult cardiomyocytes to obtain a certain level of regenerative capacity similar to that of young hearts in order to adapt to increased physiological as well as pathological workloads. It is quite obvious that a more complete list of fetal genes activated in adult patients as well as the understanding of their regenerative capacity in pediatric hearts will give a wealth of information about healing processes and help to identify disease-relevant cardiac biomarkers and therapeutic targets.

A key approach of our study was the utilization of isolated cardiomyocytes because primary cell cultures reduce the enormous complexity of a direct *in vivo* analysis. This system offers a first-class toolbox to analyze regeneration mechanisms and accelerates the screen for novel heart failure as well as circulating biomarkers by “omics” technologies [4, 12, 17]. Here, we will compare our *in vitro* results with data obtained in the developing and failing heart of patients with dilated cardiomyopathy.

## 2. Methods

### 2.1. Study Population

Patients' characteristics are shown in Table 1. Myocardial samples from four patients with aortic stenosis and preserved ejection fraction (EF > 50%) served as controls (CON) [4]. Cardiac tissue samples from six patients with end-stage heart failure due to dilated cardiomyopathy (DCM) were obtained during transplantation. Small tissue samples from 2-, 3-, and 6-month-old patients with tetralogy of Fallot were received during surgery. Moreover, hearts were collected from 3-, 8-, and 15-day-old and (adult) 12-week-old Sprague-Dawley rats after decapitation. Tissue samples were immediately flash-frozen in liquid nitrogen and kept at -80°C until use. This study complies with the Declaration of Helsinki and is approved by the respective responsible ethical committees.

### 2.2. Cell Culture, Growth, and Western Blot Analysis

Isolation, culture, and stimulation of Sprague-Dawley neonatal and adult rat cardiomyocytes were performed as previously described [18, 19]. Each experiment was performed with 20-30 hearts of 2-3-day-old neonatal pups or one 12-week-old adult heart. Cultures were performed in duplicate. The number of *n* is indicated in the figures. Adult hearts were perfused for 5 min with Ca^2+^-free Krebs-Henseleit bicarbonate buffer (KHB, pH 7.4) containing (in mM) 110 NaCl, 2.6 KCl, 1.2 KH_2_PO_4_, 1.2 MgSO_4_, 11 glucose, and 10 HEPES and gassed with 95% O_2_- plus 5% CO_2_ at 37°C. Then, after perfusion for 30 min in the same solution containing 0.04% collagenase (Worthington) and 60 *μ*M calcium, ventricles were minced in the same collagenase solution. After two washing steps at 25 g for 3 min with increasing calcium concentrations of 0.2 and 0.5 mM in KHB, myocytes were layered over a 4% BSA gradient (Sigma, fatty acid free) containing 1 mM calcium and centrifuged for 1 min at 15 g. The cell pellet was suspended then in basic medium consisting of Medium 199 with Earle's balanced salts (Sigma) without L-glutamine including 25 mM HEPES, 25 mM NaHCO_3_, 100 IU/ml penicillin, and 100 *μ*g/ml streptomycin and supplemented with 2 mM L-carnitine, 5 mM creatine, and 5 mM taurine (Sigma). Myocytes were plated on laminin- (10 *μ*g/ml; Sigma) coated chamber slides (Nunc) for fluorescence microscopy analysis and on six-well culture dishes (Falcon) for Western blots, protein synthesis determination, phase-contrast microscopy, and viability. Two hours after plating, the medium was changed, and experiments started after one day. Media were replaced every other day. Addition of 10 *μ*M 1-(b-D-arabinofuranosyl) cytosine prevented nonmyocyte growth. Similarly, neonatal ventricular cardiomyocytes were directly minced in the same collagenase solution without calcium supplementation and dissociated under shaking at 37°C for 30–45 min. Dissociated myocytes of digestion cycles 2–4 were collected in basic medium containing 1% newborn calf serum and preincubated for 1.5 h on an uncoated 148 cm^2^ dish (Falcon) to remove nonmyocytes. Nonattached neonatal myocytes were collected and then centrifuged at 1000 rpm for 5 min. The cell pellet was suspended in 1% fetal calf serum and plated on fibronectin-coated dishes (10 *μ*g/ml; PromoCell, Heidelberg, Germany) for one day. Before stimulation, cells were washed twice and cultured in basic medium as described above. Neonatal cardiomyocytes were plated at a density of 1 × 10^4^ cells/cm^2^ and adult cardiomyocytes at 0.5 (low density) or at 1.5 × 10^4^ cells/cm^2^ (high density) as indicated in the figures.

Determination of protein content and synthesis as well as DNA content of neonatal and adult cardiomyocyte cultures was reported previously in detail [18, 19]. Protein synthesis and accumulation were normalized against the DNA content as a relative measure for the cell number. At the end of the experiment, adult cardiomyocyte cultures were washed twice with phosphate-buffered saline (PBS), dissolved in lysis buffer, and processed for Western blot analysis as described previously in detail [20]. The ischemic condition was produced by reducing the O_2_ content to 1% (with 5% CO_2_) in the incubator, and cardiomyocytes were cultured in glucose-free phosphate-buffered saline (PBS).

### 2.3. Preparation of Serum, Control Samples, Morphogens, and Microvascular Endothelial Cells

Serum and tissue samples were obtained perioperatively. Cardiac microvascular endothelial cells (MVEC) were isolated from approximately 0.5 g tissue of the explanted hearts (2 patients) with DCM and grown in 20% serum on a 148 cm^2^ dish (Falcon). Microvascular endothelial cells were isolated from 6 different regions of one human myocardium. The best two cultures of each patient were used, judged by the initial number of isolated cells, their proliferation rate, and the lowest percentage of apoptotic/dying cells. Cells of the third passage were used for conditioning. Confluent cultures were washed twice, and the cell supernatant was conditioned for two days in stimulant-free pure Medium 199 with Earle's salt as described previously [18]. Proteins of this conditioned medium were concentrated 30-fold by Centricon (Millipore) with a cut-off of 5 kDa (morphogens (Morpho)). For better evaluation of specific effects of stimulants, an appropriate concentration of serum was determined giving a comparable rate of protein synthesis with Morpho at day two. Thus, Morpho was used at a 3-fold concentration and serum at a final concentration of 7.5% for experiments. For comparison, 0.2 mg/ml human serum albumin (Sigma) was added to basic medium in the controls.

### 2.4. Fluorescence Microscopy

**Figure 1:**
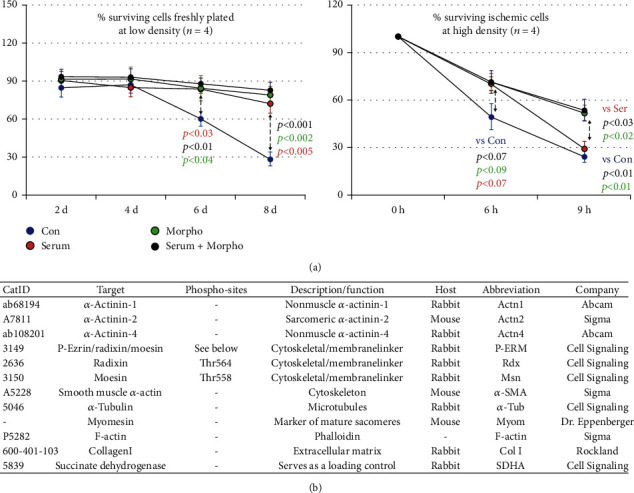
Endothelial morphogens exert protective effects on ischemic adult cardiomyocytes. Con are albumin-treated cultures. Cardiomyocytes were stimulated with serum, morphogen (Morpho), and serum plus Morpho for the indicated time in days (d) and hours (h). (a) Stimulation increases significantly the number of surviving cardiomyocytes at low density (0.5 × 10^4^ cells/cm^2^). However, under ischemic conditions, only morphogen stimulation increased the survival of cardiomyocytes (1.5 × 10^4^ cells/cm^2^). (b) List of utilized antibodies. The catalog number (Cat ID), target, phosphorylation site (phospho-site), description/function of the analyzed protein, host, used abbreviations, and distributors are listed.

Antibodies are listed in Figure 1(b) used for Western blot analysis, and tissues sections were prepared for confocal microscopy as previously described in detail [2].

### 2.5. Statistics

For statistical analysis, the unpaired *t*-test was used. *p* values less than 0.05 were considered statistically significant.

## 3. Results

### 3.1. Serum and Cardiac-Derived Endothelial Morphogens Exert Distinct Types of Adult Cardiomyocyte Remodeling

Serum and morphogens derived from cultured microvascular endothelial cells of two patients with dilated cardiomyopathy were used to analyze patterns of remodeling and dedifferentiation of cultured adult cardiomyocytes (ARC) as well as neonatal myocytes (NRC). Control cultures were treated with human serum albumin. Since we have previously demonstrated that ezrin and the reexpression of moesin monitor dynamic cellular changes, we wanted to analyze radixin (Rdx), the third component of ERM proteins, and its activated form (P-ERM) [12, 21]. Sarcomeric *α*-actinin (Actn2) identifies cardiomyocytes. The cross-striated pattern of myomesin (Myom) stain marks mature sarcomeres. Dedifferentiation is monitored by smooth muscle *α*-actin (*α*-SMA, Acta2) and nonmuscle *α*-actinin (Actn1 and Actn4) expression. These proteins are hardly expressed in cardiomyocytes of the adult tissue. Antibodies used for confocal fluorescence microscopy and Western blot analysis are listed in Figure 1(b) with corresponding distributors, short descriptions, and abbreviations.

**Figure 2:**
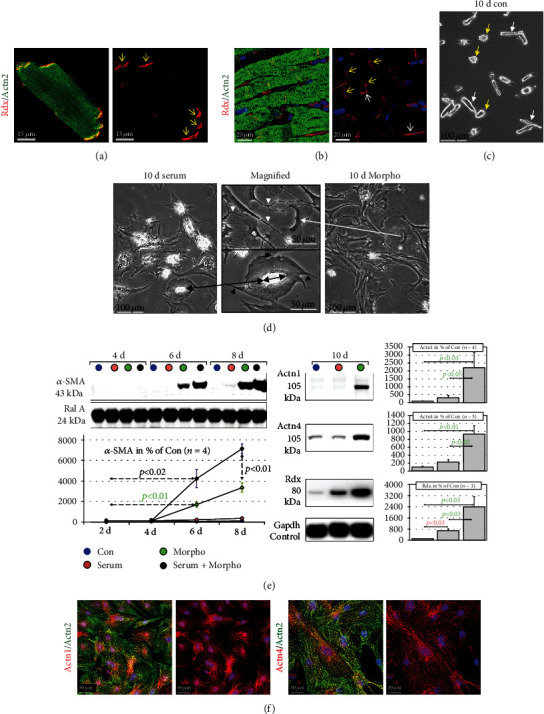
Serum and cardiac endothelial-derived morphogens induce distinct types of cardiomyocyte remodeling. Con are control cultures of adult rat cardiomyocytes treated with 0.2 mg/ml human serum albumin. Serum indicates serum stimulation, and Morpho specifies treatment with cardiac-derived morphogens (Morpho) from microvascular endothelial cells (see Methods) for the indicated time in days (d). Dedifferentiation markers are smooth muscle *α*-actin (*α*-SMA) and nonmuscle *α*-actinin-1 and *α*-actinin-4 (Actn1 and Actn4). (a) Freshly isolated adult cardiomyocytes show the typical rod-shaped phenotype *in vitro*. Radixin (Rdx, yellow arrows) staining defines the area of the intercalated disc. (b) The same applies to radixin staining of cardiomyocytes in the human myocardium (yellow arrows). Note that also some noncardiomyocytes in the interstitium are positive for radixin (white arrows). The Actn2 antibody stains sarcomeric *α*-actinin-2 in a cross-striated pattern. (c) A phase-contrast micrograph of the control group shows after 10 days that some cells still maintain a rod-shaped phenotype (white arrows) while other cardiomyocytes appear rounded (yellow arrows). (d) Phase-contrast micrographs show typical differences between the effects of serum and morphogens (Morpho) on cardiomyocytes after 10 days. The magnified image (long black arrow) shows a myocyte with an almost circular spread enlargement and little extensions after serum stimulation (black arrowheads). The double black arrow indicates the length and orientation of the original cell. In contrast, Morpho induces the formation of extremely long extensions with little lateral spreading (white long arrow and white arrowheads in the magnified image). (e) Western blot analysis reveals that Morpho induces dedifferentiation of adult cardiomyocytes. In contrast, serum stimulates very little *α*-SMA expression but enhances morphogen-induced dedifferentiation. Morphogens induce strongly the expression of Actn1 and Actn4. The expression of radixin (Rdx) reflects cardiomyocyte remodeling. Gapdh serves as a loading control. Statistical analysis is shown. (f) Confocal images show isolated neonatal cardiomyocytes (cell cultures from 3 d-old hearts).

Normal adult cardiomyocytes are rod-shaped with sharp defined intercalated discs. This phenotype is clearly visible in freshly isolated cells (Figure 2(a); Rdx, yellow arrows) as well as in myocytes of the human myocardium (Figure 2(b); Rdx, yellow arrows). In culture, unstimulated cardiomyocytes (Con) round up to a different degree in a 10-day period (Figure 2(c); yellow arrows in the control group), but still some cells maintain their rod-shaped phenotype (Figure 2(c); white arrows in the control group). Cell-cell contacts are random after plating, and no contractile activity is observed.

In contrast, serum-stimulated cardiomyocytes lose their rod-shaped phenotype and increase their surface area by spreading laterally until day 10 (Figure 2(d); long black arrow and magnified image). Cell-cell contacts are partly reestablished to cells in close vicinity by spreading, and some beating cardiomyocytes become visible. Stimulation with morphogens (Morpho) induces a completely different phenotype (Figure 2(d); long white arrow and magnified image). Myocytes show massive elongation along with the formation of multiple cellular contacts, and only some lateral spreading is recognizable. Furthermore, morphogen-stimulated cultures show multiple long and thin extensions while only few were detected in serum-stimulated myocytes (white arrowheads versus black arrowheads in magnified images). By this, morphogen-stimulated cardiomyocytes establish cell-cell contacts in the immediate and more distant vicinity.

Dedifferentiation of adult cardiomyocytes became apparent after day 4 and was strongly induced by morphogens but only mildly by serum. However, serum enhanced morphogen-induced *α*-SMA in cardiomyocytes (Figure 2(e)). Both nonmuscle *α*-actinins 1 and 4 (Actn1 and Actn4) were strongly expressed in morphogen-stimulated adult cardiomyocytes (Figure 2(e)). Since *α*-SMA as well as Actn1 and Actn4 (Figure 2(f)) is present in neonatal cardiomyocytes, their expression in adult myocytes indicates the activation of fetal pathways.

### 3.2. Opposing Effects of Serum and Morphogens on Protein Accumulation in Adult and Neonatal Cardiomyocytes

**Figure 3:**
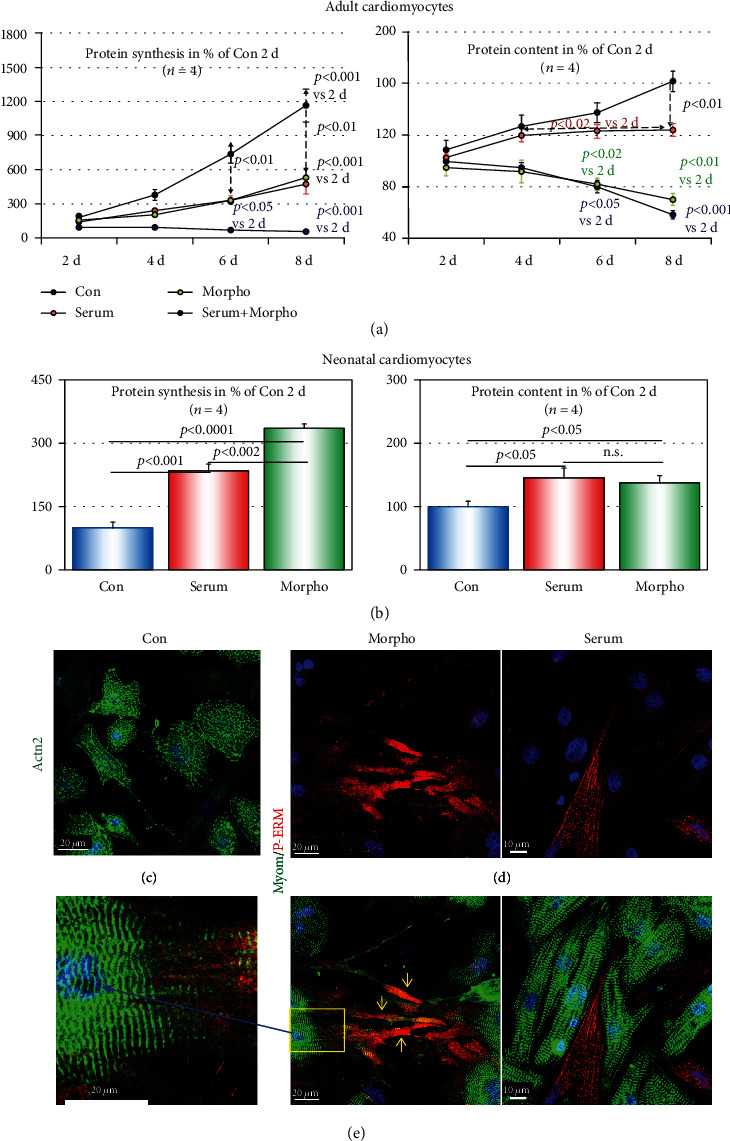
Endothelial morphogens induce atrophy of adult cardiomyocytes but hypertrophic growth of neonatal myocytes. Con are albumin-treated control cultures. Serum indicates serum stimulation, and Morpho specifies treatment with endothelial morphogens for the indicated time in days (d). All values are calculated in % of Con at day 2. (a) The time course of protein synthesis and protein accumulation (total protein content) of adult cardiomyocytes reveals significant increases after serum and serum plus morphogen stimulation. Morphogen-treated cultures show a decrease in protein content despite an elevated protein synthesis. (b) In neonatal cardiomyocytes, protein synthesis and protein content are significantly increased after stimulation for 2 days with serum or morphogen. (c, d) Confocal images of 2-day-old neonatal cardiomyocytes. (c) Unstimulated cardiomyocytes (Con) reveal a diffuse pattern of sarcomeric *α*-actinin (Actn2) and an absence of cross-striation. (d) Reestablishment of cell-cell contacts as well as the formation of mature sarcomeres (myomesin) is clearly visible after serum and morphogen stimulation. Note the long extensions in morphogen-stimulated cultures (yellow arrows). (e) Cross-striation is visible in the magnified image (blue arrow and yellow rectangle).

Here, we wanted to know the effect of serum and endothelial morphogens on protein synthesis in cultured adult cardiomyocytes by measuring [^3^H]-phenylalanine incorporation (pulse labeling) as well as protein accumulation by determination of changes in the total protein content (Figure 3(a)). Protein synthesis of the control group significantly declined steadily until day eight to 44% of the initially measured rate. In contrast, serum- and morphogen-stimulated cardiomyocytes increased continuously their protein synthesis until day eight to 480% and 534%, respectively. Combination of both stimulants showed synergistic effects (1164%). As expected, total protein content decreased in the control group to 58% while serum and serum plus morphogen increased protein content to 124% and 162%, respectively. Surprisingly, in the morphogen-stimulated group, the total amount of protein decreased significantly to 70% indicating strong protein degrading activities thereby causing atrophy of adult cardiomyocytes.

We also wanted to understand the effect of serum and morphogens on the growth of neonatal cardiomyocytes since adult cardiomyocytes develop features of neonatal cells when stimulated with morphogens. Since neonatal cardiomyocytes grow rapidly, we performed a growth analysis at day 2, a time point when adult cultured cardiomyocytes show little differences after treatment. Similar to adult cultures, serum and morphogens increased significantly the level of protein synthesis to 235% and 335%, respectively (Figure 3(b)). In contrast to adult cardiomyocytes, the elevation in protein synthesis correlates in both groups with a significant protein accumulation (145% and 138%, respectively) and indicates hypertrophic growth. In untreated control neonatal cardiomyocytes (Con), the loss of sarcomeric structure is apparent in the diffuse pattern of sarcomeric *α*-actinin staining (Figure 3(c); Actn2). Cell-cell contacts are rapidly reestablished within 2 days by elongation and spreading of stimulated cardiomyocytes (Figure 3(d)). The formation of long extensions was more pronounced in the morphogen-treated group. Furthermore, mature sarcomeres can be observed in a cross-striated pattern after myomesin staining of serum- (Figure 3(d)) and morphogen-stimulated cardiomyocytes (Figure 3(e); enlarged image).

### 3.3. Endothelial Morphogens Protect Adult Cardiomyocytes from Stress

Next, we wanted to know whether serum and morphogens exert protective effects on adult cardiomyocytes by using two different cell culture model systems. In the first model, cardiomyocytes were plated at a (low) density of 2 × 10^3^ cells/cm^2^ in order to stress cultures by keeping cell-cell contacts at a minimum. Cells were counted at the indicated days. In the damaged myocardium, a reestablishment of cell-cell contacts of surviving cardiomyocytes is necessary for the proper recovery of the contractile function. In the second model, cardiomyocytes were plated at a (high) density of 1.5 × 10^4^ cells/cm^2^ under ischemic conditions. Cultures were initially stimulated for 5 d and then kept at 0.2% O_2_ plus 5% CO_2_ in PBS for the indicated time in hours (h, ischemic model).

In the first model, cardiomyocytes were counted at the indicated time under a phase-contrast microscope and calculated in % of the originally attached number of cells (100%). In the control group, the number of attached cardiomyocytes remained relatively constant until day four and then declined to less than 30% (Figure 1(a)). In contrast, the number of attached cells in serum-, morphogen-, and serum plus morphogen-stimulated groups remained comparatively constant (73%, 79%, and 83%, respectively) indicating that growth itself is good for cells independent of the stimulus [22]. In the ischemic model, the number of attached cells decreased until 9 h to less than 30% in the control as well as in the serum-treated group. Surprisingly, the number of surviving attached cardiomyocytes was significantly higher in morphogen- (52%) and in serum plus morphogen- (54%) treated groups than in serum-stimulated cultures (Figure 1(a); 29%).

### 3.4. Cardiomyocytes in Patients with DCM Reveal Patterns of Remodeling Similar to Cultured Rat Cardiomyocytes

**Figure 4:**
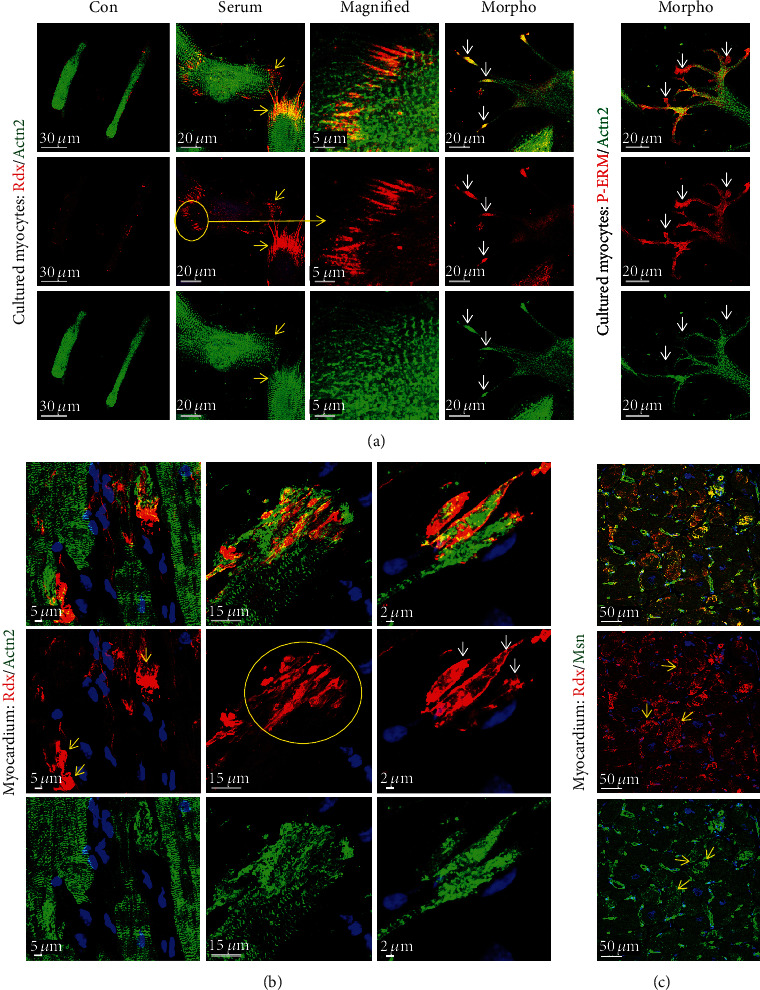
Reorganization of the intercalated disc in cultured adult cardiomyocytes resembles remodeling of ID in patients with DCM. (a) Confocal images of adult cardiomyocytes after different treatments at day 4. In control cultures (Con), radixin (Rdx) is lost at the area of the intercalated disc and cells round up at the ends. Typical cross-striation is lost. After serum stimulation, the shape of IDs appears wider (yellow arrows). Radixin accumulates in a longitudinal configuration paralleling the axis of the cardiomyocyte (yellow circle and long yellow arrow, magnified image). When long thin extensions are formed, knot-like spots at the ends can be observed (Morpho, white arrows). P-ERM indicates activation in these knot-like spots. (b) Similar to cell cultures, cardiomyocytes of the failing heart show various shapes of the ID reflecting probably different stages of remodeling (yellow and white arrows, yellow circle). The area of the ID appears wider (yellow arrows). Radixin accumulates in a longitudinal configuration in cardiomyocytes (yellow circle). Knot-like spots and extensions at the end of cardiomyocytes are clearly visible (white arrows). (c) Radixin (Rdx, red) and moesin (Msn, green) reveal also some diffuse cytoplasmic as well as membranous localization (yellow arrows).

We wondered whether activation of cultured adult cardiomyocytes reveals similarities with remodeling of myocytes in patients with end-stage heart failure. As mentioned before, cardiomyocytes are rod-shaped and radixin is mainly located at the area of the intercalated disc. Human serum albumin-treated cardiomyocytes round up at the ends within 5 days (1-day recovering time and 4-day treatment), and the typical pattern of radixin staining as well as typical cross-striations is lost but cells still maintain their rod-shaped phenotype (Figure 4(a); Con). Upon growth stimulation, radixin staining reveals various shapes of the area of the intercalated disc (Figure 4(a)). In serum-stimulated cells, the area of IDs appears wider (Figure 4(a); serum, yellow arrows) and radixin accumulates in longitudinal directions paralleling the axis of the cardiomyocyte (Figure 4(a); highlighted by the yellow circle, the long yellow arrow points to the corresponding magnified image). These patterns of radixin stains can also be found in the myocardium of patients with DCM (Figure 4(b), yellow arrows and yellow circle) indicating that these processes are evolutionary conserved. In contrast to serum, treatment with morphogens induces the formation of long thin extensions and knob-like spots at cell ends (Figure 4(a); Morpho, white arrows). These thin extensions and knob-like spots can also be observed in the human failing heart (Figure 4(b); white arrows). An overall cytoplasmic and membranous accumulation of radixin comparable to moesin in the diseased myocardium is also visible (Figure 4(c); yellow arrows) [21].

**Figure 5:**
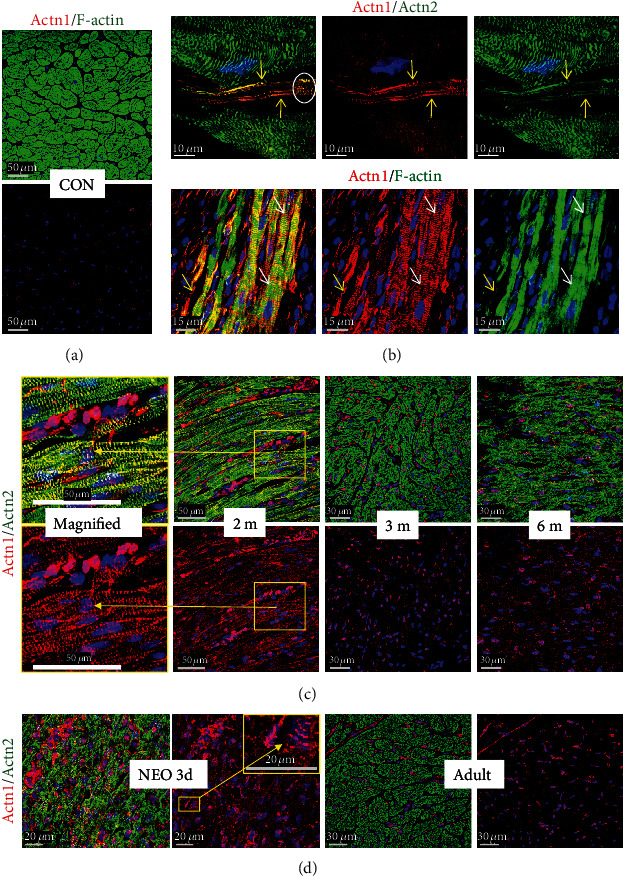
Nonmuscle *α*-actinin-1 (Actn1) is reexpressed and involved in remodeling of the area of the intercalated disc in patients with dilated cardiomyopathy. (a) Nonmuscle *α*-actinin-1 (Actn1) is completely absent in normal cardiomyocytes of the adult heart (CON). (b) Actn1 is reexpressed in myocytes of the failing human heart (HF). Note the formation of elongations (yellow arrows) from the area of the intercalated disc and the cross-striated pattern of Actn1-positive cardiomyocytes (white arrows). (c) Actn1 is present in myocytes of the myocardium of a 2-month- (2 m-) old patient with tetralogy of Fallot but absent in older infants (3 (3 m) and 6 (6 m) months). Note the cross-striated pattern of cardiomyocytes in the magnified image (yellow rectangle, long yellow arrows). (d) Actn1 is strongly expressed in cardiomyocytes of 3-day-old rat (NEO 3d) but is absent in the adult heart (Adult). Note that vessels and interstitial cells also express Actn1.

**Figure 6:**
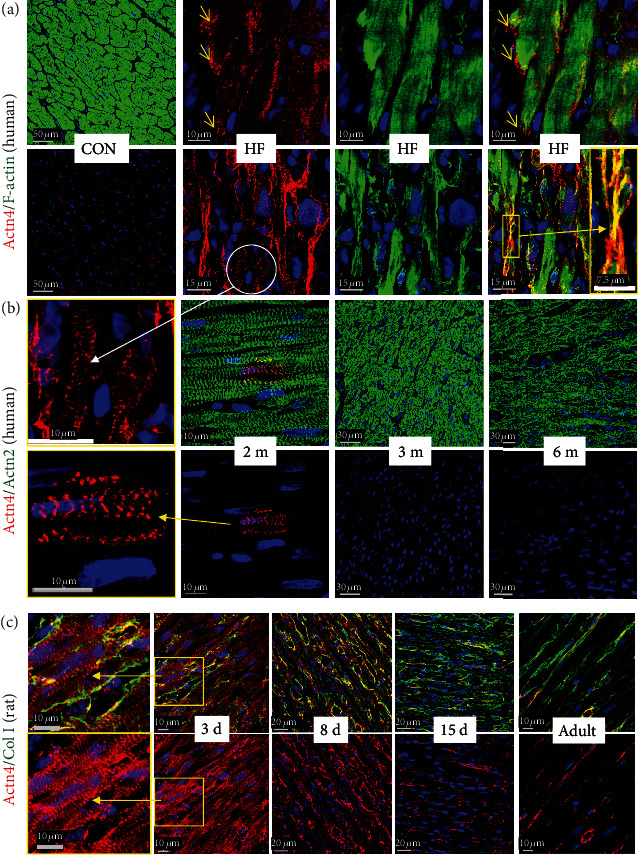
Nonmuscle *α*-actinin-4 (Actn4) is expressed and involved in remodeling of cardiomyocytes in patients with dilated cardiomyopathy. (a) Actn4 is hardly detectable in cardiomyocytes of the human adult heart (CON). In the failing heart (HF), remodeling of the area of the intercalated disc (yellow arrows) and formation of long extensions are visible (long yellow arrow, enlarged image with a yellow frame). Actn4-positive extensions of cardiomyocytes can be recognized in yellow by the overlay of green sarcomeric actinin and red Actn4. Some cross-striation (white circle and long white arrow, magnification in (b)) can be recognized. (b) Few cardiomyocytes are positive for Actn4 in the myocardium of a 2-month-old child with tetralogy of Fallot (2 m) but absent in older infants (3 (3 m) and 6 (6 m) months). (c) Actn4 is strongly expressed in cardiomyocytes of the early postnatal heart (3 days) but decreases steadily with increasing age and is absent in the adult. Note that interstitial cells and vessels express nonmuscle Actn4. Col I indicates collagen 1.

A further marker of cardiomyocyte remodeling is *α*-actinin-1 (Actn1). It is not expressed in cardiomyocytes of the normal adult myocardium (Figure 5(a); CON), but this nonmuscle *α*-actinin can be detected in long thin extensions of myocytes in the failing heart (Figure 5(b); yellow arrows). Furthermore, disruption of IDs is especially observed in areas of the myocardium with disturbed cell-cell contacts. The reexpression of the nonmuscle *α*-actinin-1 as well as the presence of sarcomeric *α*-actinin might stabilize these thin newly formed extensions. Another nonmuscle *α*-actinin, Actn4, is expressed and located at the area of the intercalated disc as well as in its extensions (Figure 6(a); small yellow arrows in HF and a long yellow arrow pointing to the magnification in a yellow rectangle).

Another surprising observation was the appearance of both nonmuscle actinins, Actn1 and Actn4, in a cross-striated pattern in cardiomyocytes of patients with heart failure (Actn1 indicated by white arrows in Figure 5(b) and Actn4 displayed by a white circle and a long white arrow pointing from Figure 6(a) to the magnified image in Figure 6(b)). While the functional role is unknown, the cross-striated presence of both nonmuscle actinins in the myocardium of newborn rats suggests a potential role in the early postnatal development of sarcomeres (Actn1 in an enlarged yellow frame image in Figure 5(d) and enlarged yellow frame images of Actn4 in Figure 6(c)). A cross-striated pattern of both nonmuscle *α*-actinins was also observed in cardiomyocytes of human infants (enlarged images of Actn1 in Figure 5(c) and Actn4 in Figure 6(b)). Furthermore, the postnatal decrease in both nonmuscle proteins in rats (Actn1 in Figure 5(d) and Actn4 in Figure 6(c)) and humans (Actn1 in Figure 5(c) and Actn4 in Figure 6(b)) during cardiac maturation indicates that these nonmuscle *α*-actinins are reactivated in the adult failing heart.

## 4. Discussion

Here, we demonstrate that remodeling of cultured adult cardiomyocytes shows similarities with myocytes of patients suffering from dilated cardiomyopathy. Remodeling of cardiomyocytes is regarded as a mechanism to cope with increased stress and involves the activation of fetal genes which gives cardiomyocytes the plasticity to adapt to environmental challenges. One of these fetal markers is nonmuscle *α*-actinin-1 (Actn1) which we previously observed in storage clusters of cardiomyocytes in the failing heart [7]. Since autophagic and ischemic cell death is a frequent event in failing hearts but colocalization of Actn1 with cell death markers was rarely observed [7], we hypothesized that nonmuscle actinins might exert certain functions and are genes expressed in developing cardiomyocytes.

Actinins (Actn1, Actn2, Actn3, and Actn4) are coded by four genes located on different chromosomes and are highly conserved between species with an almost identical length in mouse, rats, and humans. Actn3 is only expressed in the skeletal muscle but not in the heart, while Actn2 occurs in both muscle types. We also did not detect Actn3 in the myocardium of patients with heart failure by confocal microscopy (not shown). Actinins stabilize adjacent sarcomeres and anchor filaments to cell-cell contacts by cross-linking actin. Mutations in Actn1, Actn2, and Actn4 are linked to human heritable diseases [23]. Actn1 and Actn4 belong to the so-called nonmuscle *α*-actinins and reveal a high degree of sequence homology with similar actin binding properties. Despite functional similarities, diverse cell biological roles have been reported indicating that their functions are only partly overlapping. Actn1-knockout mice are not viable while Actn4-deficient mice develop a severe glomerular disease indicating that Actn1 does not compensate for the loss of *α*-actinin-1 [24]. Nuclear localization of Actn4 suggests a role in transcriptional regulation which has not been reported for Actn1 [23]. In the normal adult cardiomyocyte, nonmuscle *α*-actinins are hardly detected, but we observed a reexpression of Actn1 in the remodeling myocardium. Actn1 is reexpressed in cardiomyocytes of patients with myocardial infarction, aortic stenosis, and dilated cardiomyopathy [7, 8, 17, 25]. In mice with myocarditis and acute myocardial infarction, we observed a reexpression of this *α*-actinin in cardiomyocytes [8, 17]. Here, we detected surprisingly Actn1 in the extensions of cardiomyocytes indicating an important role of this nonmuscle *α*-actinin in the reestablishment of cellular contacts in the damaged myocardium. In addition to Actn1, we observed also a strong expression of Actn4 in cardiomyocytes of patients with dilated cardiomyopathy suggesting similar functions. However, there appears to be a timely restricted and distinct expression pattern of both nonmuscle *α*-actinins during development. Actn1 was expressed in a large number of 2-month-old human cardiomyocytes whereas few cells were positive for Actn4 at this stage. Since both nonmuscle *α*-actinins were identified in cultured neonatal rat cardiomyocytes as well as in the newborn rat heart, their expression in the adult human myocardium indicates that they are reactivated. Surprisingly, despite classification as nonmuscle *α*-actinins, staining of Actn1 and Actn4 produced a distinct cross-striated pattern reminiscent of sarcomeric *α*-actinin (Actn2) which is localized at the Z-disc. It is intriguing to speculate that these two nonmuscle *α*-actinins are involved in myofibrillogenesis and mechanotransduction in cardiomyocytes of the diseased adult myocardium as well as in the developing neonatal heart.

A further feature of remodeling is the obvious change of the architecture around the intercalated discs (IDs). IDs are the contact sites between individual cardiomyocytes and ensure mechanical and electrochemical coupling necessary for the transmission of contractile force. The function of ID in the intercellular communication and mechanical force transmission therefore suggests an important role of intercalated discs in the development of heart failure. These morphological changes have been described as membrane convolutions of IDs characteristic of the phenotype of cardiomyocytes in patients with dilated cardiomyopathy [26]. Remodeling of the intercalated disc has been furthermore observed in mice misexpressing cadherins [27], in aging mouse hearts [28], in tachypaced sheep [29], and in chronically pressure overloaded hearts of guinea pigs [30]. Staining of the ERM protein radixin indicated quite dramatic modifications in the structure of the area of IDs in patients with dilated cardiomyopathy and in cardiomyocyte cultures. Changes from accumulation of radixin into longitudinal directions paralleling the cellular axis result finally in elongated ends. We have previously demonstrated the enormous dynamic mobility of ERM proteins in cultures of adult cardiomyocytes when ezrin and radixin start to translocate from the area of the ID to different cellular locations [12]. There are a number of apparent reasons why remodeling of the area of the ID in the adult heart might be a major important adaptation to stress. The reduced ability to replace damaged myocytes by proliferation, the increasing stiffness through the rising number and maturation of sarcomeres, the oxygen-dependent energy metabolism, and the incapacity to reestablish cell contacts through migration render differentiated adult cardiomyocytes extremely vulnerable [12, 21, 26]. As a result, cardiomyocyte-cardiomyocyte contacts might be loosened or disrupted to a certain degree which can be analyzed in detail in cell cultures. However, we have at the present status also to address certain study limitations. Despite obvious localization of ERM proteins at the area of the intercalated disc, we are presently not able to distinguish whether radixin is indeed involved in remodeling of cardiomyocytes and their IDs or whether these observations are only side effects. The same applies to nonmuscle *α*-actinins. Furthermore, it is yet not clear whether the expression of Actn1 and Actn4 is indicative of an activation of the fetal gene program. Thus, further studies are needed utilizing cell cultures with siRNA knockdown of radixin and nonmuscle *α*-actinins as well as cardiomyocyte-restricted knockout mice which might also help to clarify their influence on the regenerative capacity of the diseased heart.

Generally, soluble secreted factors induce three patterns of adult cardiomyocyte remodeling in culture: first, decomposition of the area of the intercalated disc; second, formation of long extensions; and third, activation of fetal genes which can be recognized. Since all the three patterns were observed in patients, we assume that the underlying remodeling mechanisms are also valid for the development of dilated cardiomyopathy. Our cell culture data suggest furthermore that soluble secreted factors such as cytokines and growth factors play a major role in cardiomyocyte remodeling. Several important candidates have been shown to be involved in remodeling such as the interleukin-6 class of cytokines [8, 31], fibroblast growth factors [4, 32, 33], transforming growth factors [34], and insulin-like growth factors [25, 32]. However, it would be too simple to attribute the chronic and complex process of cardiac remodeling to the activity of specific cytokines, hormones, or growth factors. Cardiac pathogenesis and regeneration are rather based on a complex interplay of different factors in a spatially and temporarily restricted way. Furthermore, secreted factors often exert completely different activities pointing to distinct functions which can be derived from the effects of serum and morphogens on cardiomyocyte cultures. Serum-stimulated hypertrophy and lateral spreading with very few extensions are reminiscent of the activity evoked by fibroblast growth factors and insulin-like growth factors [25, 32, 35]. In contrast, morphogens induced expression of fetal genes, atrophy, and an intense intercellular network through the formation of multiple long extensions reminiscent of a phenotype induced by members of the interleukin-6 family [8, 31].

A further important observation is the difference in remodeling capacity of neonatal and adult cardiomyocytes in culture. Neonatal cardiomyocytes grow rapidly within 2 days and develop hypertrophy after serum as well as morphogen stimulation. In contrast, there were very few differences until day 2 in protein synthesis as well as in the protein accumulation between controls and serum- and morphogen-stimulated adult cardiomyocytes, which became only apparent after a longer culture time. Furthermore, stimulation with morphogens induced atrophy in adult cardiomyocytes while neonatal myocytes developed hypertrophy. These data indicate intrinsic variances between neonatal and adult cardiomyocytes and might partly explain the different regenerative capacity of pediatric and adult patients.

## 5. Conclusions and Summary

Our results clearly demonstrate that soluble secreted factors induce remodeling of cultured adult cardiomyocytes similar to that observed in patients with dilated cardiomyopathy. Remodeling of cardiomyocytes involved radixin relocalization and nonmuscle *α*-actinin reexpression. In culture, dedifferentiated adult cardiomyocytes develop resistance to stress but do not regain the regenerative capacity of neonatal myocytes. Since changes in protein composition and cardiac morphology are quite dramatic during chronic heart failure, it is expected that the presently known structural proteins, signaling molecules, and cascades constitute only the “tip of an iceberg.” Thus, an intense analysis of *in vitro* systems by “omics” technologies will accelerate the identification of molecules involved in cardiac regeneration and decipher hidden “survival programs” of neonatal hearts. Determination of these factors and their individual regenerative potential are of particular importance for a biomarker-guided therapy in adults.

## Figures and Tables

**Table 1:** Clinical data of the analyzed patients. Six patients (3 females and 3 males) developed the phenotype of dilated cardiomyopathy (DCM) without signs of coronary heart disease (CHD) who had undergone heart transplantation (HTX). Left ventricular ejection fraction (LVEF) was lower than 20%. Four patients have aortic stenosis (AoSt) and preserved ejection fraction (EF > 60%; 2 males and 2 females). Three pediatric patients with tetralogy of Fallot (ToF) served for comparison. NYHA: New York Heart Association; PCI: percutaneous coronary intervention; CABG: coronary artery bypass grafting; AK-OP: aortic valve surgery; MK-OP: mitral valve surgery; bivICD: biventricular implantable cardioverter-defibrillator; ACE: angiotensin-converting enzyme; AT1: angiotensin II receptor; ASS: acetylsalicylic acid; CRP: C-reactive protein; LDH: lactate dehydrogenase; SGOT: serum oxaloacetic transaminase; SGPT: serum glutamic pyruvic transaminase; CK: creatine kinase; NT-pro-BNP: N-terminal probrain natriuretic peptide; PTT: activated and partial thromboplastin time; INR: international normalized ratio; HIV: human immunodeficiency virus; LVESD: left ventricular end-systolic diameter; LVEDD: left ventricular end-diastolic diameter; PVR: pulmonary vascular resistance; PCWP: pulmonary capillary wedge pressure.

Demographic and risk factors	DCM (mean ± s.e.m.)	AoSt (mean ± s.e.m.)	ToF (mean ± s.e.m.)
Number of patients	6	4	3
DCM	6	0	0
CHD	0	0	0
Etiology of DCM	3xi.p., 2xfam, 1xpp	0	0
Age	54 ± 4 years	65 ± 10 years	2, 3, and 6 months
Gender	3 females, 3 males	2 females, 2 males	1 females, 2 males
NYHA class	3.8 ± 0.2	3	0
Body height (cm)	170 ± 5	170 ± 5	64 ± 2
Weight (kg)	64 ± 4	84 ± 9	6.6 ± 0.4
Prior myocardial infarction	0	0	0
Prior PCI or CABG	0	0	0
Prior smoking	2	1	0
Prior hypertension	1	3	0
Prior COPD	1	0	0
Prior diabetes mellitus	0	1	0
Thrombocytopenia	0	0	0
Prior hypercholesterolemia	1	1	0
Pre-CABG	0	0	0
Pre-HTXLVAD	0	0	0
Pre-HTXAK-OP	1	0	0
Pre-HTXMK-OP	1	0	0
Pre-HTXbivICD	5	0	0
Cardiac arrhythmia	3	1	0
Comedication			
Beta blocker (%)	6	4	3
ACE inhibitor and/or AT1 antagonist (%)	4	4	0
Diuretic (%)	6	2	0
Digitalis (%)	0	1	0
Aldosterone antagonist (%)	4	1	0
Sildenafil 20 mg (%)	2	0	0
ASS (%)	0	2	0
Amiodarone (%)	2	0	0
L-Thyroxin (%)	1	1	0
Statins (%)	1	0	0
Laboratory parameters			
Sodium (mmol/l)	137 ± 1	139 ± 0.6	138 ± 0.7
Potassium (mmol/l)	4.5 ± 0.2	4.6 ± 0.4	4.5 ± 0.3
Creatinine (mg/dl)	1.6 ± 0.2	1 ± 0.2	0.2
Hemoglobin (g/dl)	9.7 ± 0.3	12.8 ± 1.4	12.5 ± 0.1
Hematocrit (%)	29.2 ± 1	39.2 ± 4.2	0.37
CRP (mg/l)	1.2 ± 0.4	2.8 ± 2.7	<0.5
Leukocytes (Ts/*μ*l)	10.9 ± 1.3	10.1 ± 1.4	15.3 ± 4
Total cholesterol (mg/dl)	171 ± 19	217 ± 32.5	n.d.
LDHU (l)	403 ± 153	286 ± 30	306 ± 13.3
SGOT (IU/l)	72 ± 41	57 ± 7.3	33 ± 1.2
SGPT (IU/l)	24.2 ± 8.4	67 ± 24	27 ± 4.7
CKU (l)	361 ± 263	75 ± 19	307 ± 144
NT-pro-BNP (pg/ml)	6363 ± 2783	1663 ± 1260	57 ± 21
Quick (thromboplastin time) (%)	80.7 ± 15.2	75 ± 11.7	96 ± 6.7
PTT (s)	31 ± 2	31 ± 2.2	34 ± 1.8
INR	1.4 ± 0.2	1.3 ± 0.2	1 ± 0.1
Hepatitis B & C, HIV	0	0	0
Echo characteristics			
LVEF (%)	19.2 ± 2.4	64 ± 2.4	n.d.
LVESD (mm)	57 ± 4	31 ± 2.2	n.d.
LVEDD (mm)	65 ± 4	53 ± 2.3	n.d.
Hemodynamic parameters			
Cardiac index (l/min∗m)	23.8 ± 4.1 (1.8 ± 0.2)	n.d.	n.d.
PVR (dynes∗s/cm^5^)	190 ± 22	n.d.	n.d.
PCWP (mmHg)	26.3 ± 3	n.d.	n.d.
O_2_ saturation (%)	46.7 ± 2.5	n.d.	n.d.

## Data Availability

The data as well as the images that support the findings of our study are available from the corresponding author Dr. Thomas Kubin (t.kubin@kerckhoff-klinik.de) upon reasonable request.
